# Immunomodulation in Children: The Role of the Diet

**DOI:** 10.1097/MPG.0000000000003152

**Published:** 2021-04-16

**Authors:** Elvira Verduci, Jutta Köglmeier

**Affiliations:** ∗ Department of Pediatrics, Ospedale dei bambini V. Buzzi; † Department of Health Science, Università degli Studi di Milano, Milan, Italy; ‡ Department of Paediatric Gastroenterology, Great Ormond Street Hospital for Children NHS Foundation Trust, London, United Kingdom.

**Keywords:** arginine glutamine, bioactive peptides, conjugated linoleic acid, docosahexaenoic, diet, immune system, inflammation, nutrients, protein, trace elements, vitamins

## Abstract

Immune maturation and response to inflammation depend on good nutritional status. Protein and amino acid deficiencies can compromise innate and adaptive immune functions, particularly following injury or during illness. Dietary omega-3 long-chain fatty acids, prebiotics and micronutrients are beneficial to the immune system. A complex interplay exists between diet, microbiome, and epigenetic factors. The effect of single nutrients on immune function may hence be difficult to study. Well-designed intervention studies, investigating the effects of whole dietary pattern on the immune system, are needed.

## Abstract 2

Supplemental Digital Content is available in the text

**What Is Known/What Is New**

**What Is Known**

- Several macro and micronutrients influence the immune system.
- A complex interplay exists between diet, microbiome and epigenetic factors.
- The effect of single nutrients on immune function may be difficult to study.

**What Is New**

- Foods rich in arginine, glutamine, bioactive peptides, docosahexaenoic, prebiotics, zinc, iron, copper, selenium, and vitamins (D, A, E, group B, C) should be offered to stimulate immune function in children.
- Nutrition counselling should start early in life, emphasizing the importance of foods with immune-modulating properties, promoting healthy eating.
- More information regarding the optimal dietary intake (and blood/plasma levels) to achieve an immunoregulatory action of these nutrients are desirable;
- Well-designed intervention studies, investigating the effects of whole dietary pattern on the immune system, are needed.

A functioning immune system is essential to maintain health. An optimal nutrient intake is shown to modulate immune maturation and response to inflammation, likely mediated by gut microbiota composition and function and through epigenetic mechanisms (Fig. 1). On the other hand, clinical conditions characterized by acute on chronic inflammation may increase requirements for nutrients and promote a nutrient-wasting/standing catabolic state (2). In turn, malnutrition can contribute to infection susceptibility, and infections contribute to malnutrition, in a vicious cycle (Fig. 1).

**FIGURE 1:**
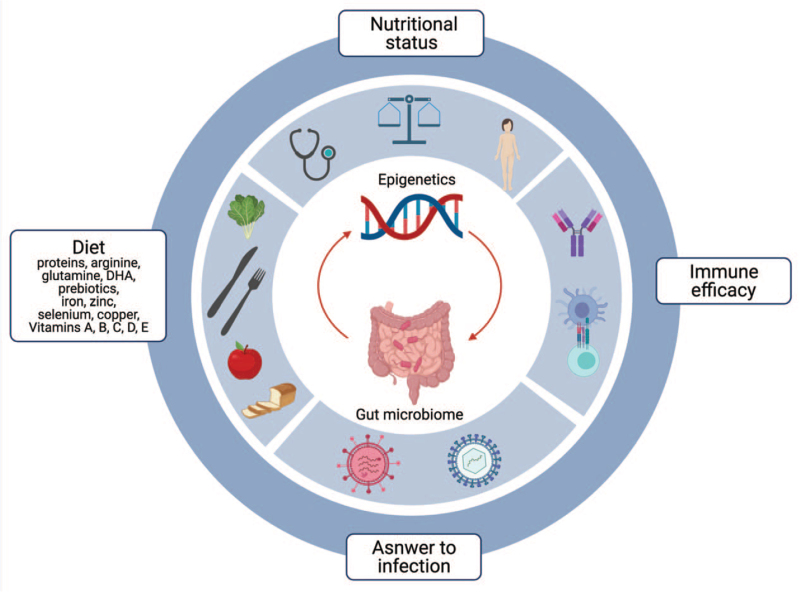
Visual representation of the complex relationship between nutritional state, response to inflammation and immune maturation (this image needs to be in-print version).

The present paper aims to review macro and micronutrients in the diet, which have an action on the immune system and to advise on a diet with “immunomodulating” properties for children.

## METHODS

To prepare the present review Medline and Cochrane Library were used as sources of information. A search for Cochrane reviews, other systematic reviews, and meta-analysis as evidence-based clinical practice guidelines was performed (Supplemental Digital Content, *http://links.lww.com/MPG/C335*).

## RESULTS AND DISCUSSION

An imbalanced diet might result in suboptimal immune function and can be caused by insufficient intake or absorption of macro- and micronutrients. Tables, Supplemental Digital Content (*http://links.lww.com/MPG/C336*, *http://links.lww.com/MPG/C337*) report the European Food Safety Authority population reference intakes (PRIs) and adequate intakes (AIs) and the main dietary sources of each nutrient, considered in the present review, with immunomodulatory effect.

### Role of Proteins

A condition of protein-energy malnutrition (PEM) has been associated with immune dysfunction and higher risk of infection (2). Indeed, malnutrition is related to mucosal and skin barrier alterations. Protein deficiency can lead to impaired gastrointestinal barrier function, loss of lymphoid tissue and altered intestinal microbiota. As a consequence susceptibility to enteric pathogens is increased, impacting on the high rates of cell proliferation and DNA replication in the intestinal epithelium (3).

In addition, protein deficiencies impact on hematopoietic and lymphoid organs (cellularity and functions of thymus, bone marrow, spleen, lymph nodes, gut-associated lymphoid tissue or GALT) and compromise innate and adaptive immune functions (3).

Arginine is essential for adequate functioning of the cardiovascular and immune system, and wound healing (4). Postoperatively arginine stimulates the response of macrophages/monocytes to antigens and is a substrate for immune cells responsible for functioning of T lymphocytes and macrophages (5). Arginine-enriched parenteral nutrition improves survival following peritonitis by normalizing nuclear factor kappa B (NF-κB) activation in peritoneal resident and exudative leukocytes (6). Moreover enteral nutrition containing arginine, omega-3 polyunsaturated fatty acids (PUFA) and nucleotides supports the immune system. Eicosapentaenoic (EPA) and docosahexaenoic (DHA) suppress the systemic inflammatory response by reduced expression of the arginine degrading arginase (7). There are few RCTs on l-arginine supplementation in children. Reduced nitric oxide concentration may be a possible mechanism of necrotizing enterocolitis (NEC). A recent Cochrane systematic showed that arginine supplementation may prevent NEC in preterms (8), but more data are needed (8).

Glutamine also plays an important role in normal function of the immune system (9). It is essential for lymphocyte proliferation (by epigenetic mechanisms), and cytokine production (interleukin [IL]-6, interferon [IFN], and TNF), macrophage and neutrophils activities (10). In healthy patients, eating a balanced diet, glutamine supplementation is not necessary to increase the effectiveness of immune system; however, in the catabolic state or in a low protein/glutamine intake from the diet, glutamine supplementation could be required (2,9)

Therefore, it is important to ensure an appropriate protein intake (according to PRI for sex and age (1)), in particular during the period characterized by a rapid growth, especially the first years of life, and during puberty (11).

Finally, different bioactive peptides with immunomodulatory actions have been explored. Most studied concentrate on dairy proteins (milk and fermented milk), but vegetable plant- and animal-derived proteins (rice, egg) have also been evaluated (12). Peptides may be absorbed in the intestine, interact with macrophages, dendritic cells or B lymphocytes, influencing the production of cytokines and antibodies.

Focusing on this topic could provide future tools to generate new functional foods with beneficial effects on health from natural products.

### Role of Lipids

Dietary lipids are an important source of energy for malnourished children because of their high caloric dense n-6 (omega-6) and n-3 (omega-3) polyunsaturated fatty acids (PUFAs). The impact of dietary PUFA on the immune system is well known (13). DHA in particular and omega-3 PUFAs in general, have shown to modulate both innate and adaptive immunity (14). Eicosanoids, metabolites derived from long-chain PUFA (LCPUFA), have specific actions. DHA can inhibit the inflammatory response by mediators (resolvins, protectins, and maresins) (15). EPA and DHA derived mediators named E- and DA-series resolvins (RvE and RvD) together with the DHA derived neuroprotectin D1 appear involved in inflammation resolution (16). RvE and RvD resolve inflammation similarly, whereas (neuro-) protectins have immunoregulatory action (17). The DHA-metabolites maresins (18) are strong anti-inflammatory mediators with similar effectiveness to RvE and RvD (18). Data about the immunomodulatory and anti-inflammatory actions of resolvins and maresins in humans are scarce (14). An adequate daily intake of EPA and DHA in children should be 250 mg for children from 2 years onwards, in one or more servings (as in adults with respect to primary cardiovascular disease prevention). In addition 100 mg of preformed DHA should be consumed during the first 2 years of life (19). The main dietary sources of DHA are oily fish (20) (Tables, Supplemental Digital Content, *http://links.lww.com/MPG/C336*, *http://links.lww.com/MPG/C337*) but fish high in mercury should be limited to one serving per week to avoid poisoning (21).

The conjugated linoleic acid (CLA), a trans fatty acid (FA) produced by bacterial biohydrogenation of FAs in the rumen of ruminant livestock like cows and therefore present in cow's milk, dairy products and breast milk, can modulate immune function (22). In particular, CLA acts on the production of cytokines, eicosanoids (prostaglandins, leukotrienes), and nitric oxide and is involved in the inhibition of eosinophilic cationic protein and the expression of peroxisome proliferator-activated receptor gamma (PPARγ). It has also shown to have a protective effect on the development of atopic manifestations (22) in infants.

### Role of Carbohydrates

#### Dietary Prebiotics

Dietary prebiotics are typically non-digestible carbohydrates (23), which influence the composition and activity of the intestinal microbiota (24). Several studies demonstrate that consuming a wide variety of carbohydrates with prebiotic activity increases the number of bacteria beneficial to human health (25,26).

The mechanism by which prebiotics have a beneficial effect on the functioning of the immune system has not yet been fully understood. Some mechanisms are hypothesized (26):

- increased production of short-chain fatty acids (SCFAs), such as propionic acid acting on hepatic lipogenic enzymes;
- increased production of SCFAs (especially butyric acid), which act as genetic transcription factors;
- modulation of mucin production;
- increased numbers of lymphocytes and/or leukocytes in GALT and peripheral blood;
- stimulation of phagocytic function of the inflammatory macrophages by increased secretion of immunoglobulin A by the GALT.

Carbohydrates with potential prebiotic activity are found in edible plants (27) (Tables, Supplemental Digital Content, http://links.lww.com/MPG/C336, http://links.lww.com/MPG/C337).

### Micronutrients

Minerals and vitamins play a crucial role both in innate and adaptive immune response, and adequate intake/serum levels are essential to reduce susceptibility to infections (28); however, in conditions associated with a systemic inflammatory response, interpretation of vitamin and trace element concentrations in blood can be misleading in clinical practice (29). The main effects of vitamins on the immune system are summarized in Table 1.

**TABLE 1:** Effects of Vitamins on Immunomodulation

Vitamins	Immunomodulatory effects
Vitamin A	Vitamin A increases the mechanistic defense and supports antigen non-specific immunity functions of the mucosa by enhancing mucin secretion. Vitamin A has also shown the ability to regulate differentiation, maturation, and function of several mediators of the immune system, such as macrophages and neutrophils (52).
Vitamin B6	It is involved in immune function with interleukin-2 (IL-2) production (53).
Vitamin B9 (folic acid) and B12	Vitamin B12 has been shown to play a particularly important role for the cytotoxic immune response mediated by both, natural killer (NK) cells and CD8^+^ T cells by upregulating these cells. Deficiency of vitamin B12 leads to trapping of tetrahydrofolic acid (THF) in its methylated form and the accumulation of methyl-THF leading to a number of health impairments. Maintaining the balance of the two vitamins is therefore important also with regards to the immune response. It was shown that it has particular effects on NK cells and cytotoxic CD8^+^ lymphocytes (54). Blood levels of vitamin B12 above 221 pmol/L (>300 ng/L or pg/mL) are considered normal.
Vitamin C	Many aspects of the proper functioning of the immune system depend on vitamin C, including growth and function of both innate and adaptive immune cells, phagocytosis and microbial killing, antibody production and supportive epithelial barrier function (55).
Vitamin D	In innate immunity, vitamin D exerts a stimulatory role: it enhances antimicrobial peptides production, such as cathelicidin and β-defensin, by monocytes and macrophages. It also promotes autophagy, chemotactic and phagocytic abilities of innate immune cells. Moreover, vitamin D acts on the adaptive immune system, with a general inhibitory effect both on T and B cells. Naïve CD4^+^ T cells can either differentiate into T_H_1, T_H_2, T_H_17, and Treg cells. Vitamin D inhibits dendritic cells differentiation and maturation, thus favoring a more tolerogenic state (56). The term hypovitaminosis D refers to serum 25 (OH)D levels <30 ng/mL.
Vitamin E	Vitamin E has several important effects on the immune system, being involved in phagocytosis, T cell proliferation and differentiation, and antibodies production.Furthermore, by acting as a scavenger of reactive oxygen species, vitamin E prevents the propagation of free radicals, which are particularly harmful to polyunsaturated fatty acids in membrane phospholipids and blood lipoproteins.During immune reactions, it also protects cells and functional components such as proteins and fatty acids from damage caused by defense mechanisms against pathogens (57)

Dietary sources are present in Tables, Supplemental Digital Content, *http://links.lww.com/MPG/C336*, *http://links.lww.com/MPG/C337*.

### Trace Elements

#### Zinc

Normal zinc levels are important for the maintenance of the immune system (3).

The normal development and function of innate cell-mediated immunity, neutrophils, and natural killer cells are closely related to zinc homeostasis. Indeed, macrophages, phagocytosis, intracellular killing, and cytokine production are compromised by zinc deficiency (30). T- and B-cell growth and function are also affected by low zinc levels through promotion of T_H_1 cell differentiation and T_H_1 cell responses by increasing IL-2, IFN-, and IL-12Rb2 expression levels. Additionally, zinc regulates the release of proinflammatory cytokines such as IL-1, IL-6, and TNF-α by innate immune cells (31,32).

Zinc deficiency is associated with diarrhea and respiratory infections (33). Zinc had a significant role in preventing respiratory infections among children of 2–60 months in low- and middle-income countries (34). A recent systematic review conducted on 522 Iranian children ages 6 months to 3 years showed that zinc supplementation reduced the severity and duration of respiratory infections (35).

Main dietary sources of zinc are plant foods such as whole grains and some nuts, and animal-derived foods like red meat, fish and cheese (Tables, Supplemental Digital Content, *http://links.lww.com/MPG/C336*, *http://links.lww.com/MPG/C337*).

#### Copper

Copper is bactericidal, essential for cell-mediated immunity and specific antibody formation (37). Indeed it has an important role in the maintenance of proper function of the immune system. Macrophages (eg, copper accumulates in phagolysosomes of macrophages to combat certain infectious agents), T helper cells, B cells, neutrophils, and natural killer cells, are significantly supported by copper. It is therefore essential for cell-mediated immunity and specific antibodies generation (36).

Children suffering from copper deficiency are susceptible to bacterial infections (37). Low levels were associated with an increased susceptibility to respiratory infections in Chinese children of 2–6 years old in a meta-analysis of 11 studies (38). Copper alongside zinc supplementation has been studied in Indian children ages 6–59 months with diarrhea but was not found to be cost-effective (39).

Liver and fish are rich in copper; small amounts are found in aged cheeses. Nuts and cocoa are a vegan alternative source (Tables, Supplemental Digital Content, *http://links.lww.com/MPG/C336*, *http://links.lww.com/MPG/C337*).

#### Selenium

Selenium is incorporated into selenoproteins required for normal immune function (40). It exerts its pivotal anti-inflammatory effects through the mitogen-activated protein kinase (MAPK)-, NF-κB-, and PPAR-dependent regulation of proinflammatory mediators (41).

Selenium deficiency has been associated with sepsis in intensive care patients. Preterm infants are prone to low levels and supplementation decreases the risk of nosocomial sepsis (42)

Liver, meat and fish all contain selenium (Tables, Supplemental Digital Content, *http://links.lww.com/MPG/C336*, *http://links.lww.com/MPG/C337*). Low selenium intake is found in many areas of Europe due to low levels in agricultural soil (43).

#### Iron

The proliferation and maturation of immune cells, in particular lymphocytes, is iron-dependent (44). The release of reactive oxygen species has been shown to be promoted by Intracellular iron activating NF-kB. The production of antimicrobial peptides by macrophages is promoted by hypoxia-inducible factor-1 alpha (HIF-1α), an iron-dependent transcription factor. Iron supplementation in iron-deficient patients, has shown peripheral blood mononuclear cells with increased TNF-α, IL-6, and IL-10 mRNA expression. The proliferation of human B and T lymphocytes was also reduced by TfR1-blocking antibodies (45,46).

Iron deficiency is common and results in iron deficiency anemia associated with a decrease in immune response to infections, fatigue and response to metabolic stress, reduced cognitive functions and impaired growth (47,48). Iron deficiency is diagnosed when serum ferritin levels are below 12 μg/L for children less than 5 years, or below 15 μg /L for those 5 years and over (49).

Combined supplementation of iron and vitamin A reduced the incidence of diarrhea- and respiratory-related illnesses in Chinese preschool children (50). It is important to mention that serum ferritin, as a biomarker to evaluate the iron status, can be only be used in noninflamed patients (44).

Food contains heme and non-heme iron. The former one is found in meat and contributes to 20% of the intake. The bulk originates from non-heme iron originating in vegetables and dairy products but is less well absorbed (51). Vitamin C consumption alongside iron improves absorption (51).

Meat, egg, grains, legumes, and some vegetables are rich in iron (Tables, Supplemental Digital Content, *http://links.lww.com/MPG/C336*, *http://links.lww.com/MPG/C337*).

## CONCLUSIONS AND LEARNING POINTS

**FIGURE 2:**
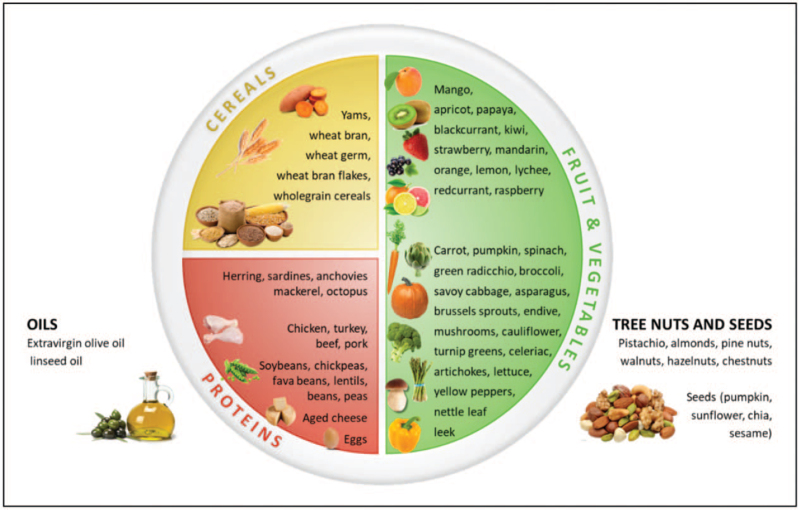
Visual representation of foods with potential immune-modulatory properties.

- Several macro- and micronutrients influence the immune system.
- A complex interplay exists between diet, microbiome and epigenetic factors. The effect of single nutrients on immune function may hence be difficult to study. Indeed, information regarding the optimal dietary intake (and blood/plasma levels) to achieve an immunoregulatory action of these nutrients are lacking;
- Well-designed intervention studies, investigating the effects of whole dietary pattern on the immune system, are needed.
- Infants should be breastfed as long as possible (52) and foods rich in arginine, glutamine, bioactive peptides, DHA, prebiotics, zin, iron, copper, selenium and vitamins (D, A, E, group B, C) should be offered when solids are introduced.
- Nutrition counseling should start early in life emphasizing the importance of foods with immune-modulating properties, promoting healthy eating (Fig. 2).
- Vitamin and trace elements biomarkers should be interpreted in relation to the overall clinical condition and history of the individual patient before considering a possible supplementation.

## Supplementary Material
