# Supplementary material for: Immunomodulation in Children: The Role of the Diet
Source: J Pediatr Gastroenterol Nutr. 2021 Apr 16;73(3):293–8. doi: 10.1097/MPG.0000000000003152 (PMC9770123; doi:10.1097/MPG.0000000000003152)
Supplement: Supplementary file 1 [file jpga-73-293-s001.docx]

The following search terms were used (Medline and Cochrane)

((Nutrition [MeSH Terms]) OR (diet [MeSH Terms]) OR (dietary pattern [MeSH Terms]) OR (macronutrients [MeSH Terms]) OR (micronutrients [MeSH Terms]) OR (proteins [MeSH Terms]) OR (aminoacids [MeSH Terms]) OR (lipids [MeSH Terms]) OR (DHA [MeSH Terms]) OR (dietary prebiotics [MeSH Terms]) OR (trace elements [MeSH Terms]) OR (vitamins [MeSH Terms]) )AND ((immune function [MeSH Terms]) OR (immune system [MeSH Terms]) OR (immunomodulation [MeSH Terms]) OR (immunity [MeSH Terms])) with limits to “Full text”, “Books and Documents”, “Review”, “Systematic Reviews” and from 2010 to 2020.
