# Supplementary material for: Immunomodulation in Children: The Role of the Diet
Source: J Pediatr Gastroenterol Nutr. 2021 Apr 16;73(3):293–8. doi: 10.1097/MPG.0000000000003152 (PMC9770123; doi:10.1097/MPG.0000000000003152)
Supplement: Supplementary file 2 [file jpga-73-293-s002.docx]

Table 1 (reporting Population Reference intakes and Adequate Intake for nutrients with immunomodulatory properties)

| ***Population Reference intakes (PRIs)** and Adequate Intake ( AIs)  for nutrients with immunomodulatory properties | | | | |
| --- | --- | --- | --- | --- |
| ***Age group*** | 6-12 mo | 1-3 y  4-6 y  7-10 y | 11-14y males  15-17y males | 11-14y females  15-17y females |
| ***Nutrient*** |  |  |  |  |
| Proteins  (g/kg/day) | **1.32** | **1.00**  **0.94**  **0.99** | **0.97**  **0.93** | **0.95**  **0.90** |
| DHA  (mg/day)  EPA + DHA (mg/day) | 100 | 250  250 | 250  250 | 250  250 |
| Dietary Fibre (g/day) |  | 10  14  16 | 19  21 | 19  21 |
| Zinc*  (mg/day) | **2.9** | **4.3**  **5.5**  **7.4** | **10.7**  **14.2** | **10.7**  **11.9** |
| Copper  (mg/day) | 0.4 | 0.7  1.0  1.0 | 1.3  1.3 | 1.1  1.1 |
| Selenium  (µg/day) | 15 | 15  20  35 | 55  70 | 55  70 |
| Iron  (mg/day) | **11** | **7**  **7**  **11** | **11**  **11** | **11**  **11** |
| Vitamin A  (μg RE/day) | **250** | **250**  **300**  **400** | **600**  **750** | **650**  **650** |
| Vitamin B6  (mg/day) | 0.3 | **0.6**  **0.7**  **1.0** | **1.4**  **1.7** | **1.4**  **1.6** |
| Vitamin B9 (folic acid)  (µg DFE**/day) | 80 | **120**  **140**  **200** | **270**  **330** | **270**  **330** |
| Vitamin B12 (µg/day) | 1.5 | 1.5  1.5  2.5 | 3.5  4.0 | 3.5  4.0 |
| Vitamin C  (mg/day) | **20** | **20**  **30**  **45** | **70**  **100** | **70**  **90** |
| Vitamin D (µg/day) | 10 | 15  15  15 | 15  15 | 15  15 |
| Vitamin E (α-Tocopherol) (mg/day) | **5** | **6**  **9**  **9** | **13**  **13** | **11**  **11** |

**Dietary Folates Equivalents . µg DFE = µg food folate + (1.7 x µg folic acid )
