# Supplementary material for: Immunomodulation in Children: The Role of the Diet
Source: J Pediatr Gastroenterol Nutr. 2021 Apr 16;73(3):293–8. doi: 10.1097/MPG.0000000000003152 (PMC9770123; doi:10.1097/MPG.0000000000003152)
Supplement: Supplementary file 3 [file jpga-73-293-s003.doc]

Table 2 (reporting mean nutrients’ content in 100g of raw, unprocessed food)

| **Nutrients content in 100g of raw food** | | | | | | | | |
| --- | --- | --- | --- | --- | --- | --- | --- | --- |
|  | **Meat** | **Fish** | **Dairy products**  **And Hen’s egg** | **Legumes** | **Cereals** | **Fruits** | **Vegetables** | **Other** |
| **Arginine** | Bresaola 2048mg  Pork ham 1675mg  Chicken breast 1626 mg  Turkey breast 1536mg |  |  | Peanuts 3680mg  Soybeans *3153mg  Fava beans * 2411mg  Chickpeas* 1939mg  Lentils * 1903mg |  |  |  | Pine nuts 6229mg  Pistachio 2266mg  Almonds 2171mg  Hazelnuts 2042mg  Walnuts 2003 mg  Pumpkin seeds *5353mg  Sesame seeds* 2630mg  Sunflower seeds* 2403mg  Chia seeds* 2143mg |
| **Glutamine** | Lamb 4660 mg  Turkey 4660 mg  Breast Chicken 4572 mg |  |  | Milk 6004 mg  Asiago cheese 7020 mg  Eggs 1508 mg |  |  |  | Pine nuts 6740 mg  Pistachio 5808 mg |
| **Peptides** |  |  | Eggs (ovalbumin, ovomucin)  Milk (𝛼s1-casein, 𝛽-lactoglobulin) |  | Rice |  |  |  |
| **ALA**  **(α-linolenic acid)** |  |  |  |  |  |  |  | Linseed 17.10g  Linseed oil  Soy oil 7.6g  Chia seeds 17.83g  Walnuts 6.64g |
| **DHA** |  | Mackerel 1.26g  Sardines 1.16g  Herring 1.01g |  |  |  |  |  |  |
|  |  |  |  |  |  |  |  |  |
| **CLA**  **(conjugated linoleic acid)** | Beef 0.77 mg  Lamb 0.55 mg |  | Milk 0.07 mg  Permigiano Cheese 0.38 mg  Eggs 1.06 mg |  |  |  |  | Cocconuts Oil 1.6 mg  Sunflower Oil 49.89 mg |
| **Dietary fiber** |  |  |  | Chickpeas flour 13.8g  Soyseeds 11.9g  Peanuts 10.9g  Beans 10.6g  Peas 5.2g  Fava beans 5.1g | Wheat bran 39.6g  Wheat bran flakes  17.3g  Wheat germ 15.6g  Barley flakes and flour 14.8g  Durum wheat 12.6g  Oat 10.6g  Whole-wheat flour 10g | Coconut 12.2g  Dried fruit 11.9g  Blackcurrant 7.8g  Redcurrant 7.4g  Raspberries 7.4g  Quince 5.9g | Mushrooms (average) 6g  Brussels sprouts 5.2g  Celeriac 5.1g  Nettle leaf 4.1g  Broccoli 3.1g  Carrots 3.1g  Savoy cabbage 3.1g | Linseed 34.8g  Almonds 12.7g  Chestnut flour 10.9g  Pistachio 10.6g  Hazelnuts 8.1g  Walnuts 6.2g  Chestnut 4.7g  Pine nuts 4.5g  Tempeh 4.1g |
| **FOS**** |  |  |  | Green peas  Lentils  Soy beans | Barley  Rye bread  Rye crackers  Pasta  Gnocchi  Couscous  Wheat bran  Wheat bread  Oats | Banana  Custard apples,  Rambutan  Grapefruit Pomegranate  Dried fruit (eg. dates, figs) | Onion  Garlic  Jerusalem Artichokes  Asparagus  Beetroot  Fennel bulb  Snow peas  Sweetcorn  Savoy cabbage |  |
| **GOS**** |  |  |  | Beans  Red kidney beans  Chickpeas  Split peas |  | Nectarines  White peaches Persimmon  Tamarillo  Watermelon |  | Cashews  Pistachio |
| **Inulin^┼┼^** |  |  |  |  | Rye  Barley  Wheat |  | Onion  Artichoke  Chicory Dandelion  Leek  Garlic  Yacon |  |
| **Zinc** | Beef meat 5mg | Octopus 5.1mg  Anchovies 4.2mg | Aged cheese (average) 4.5mg  Parmesan or Grana cheese 4mg |  | Wheat germ 17mg  Wheat bran 16.2mg  Oat 4mg  Wheat flour 4mg |  |  | Linseed 7.8mg  Pine nuts 6.5mg  Cashews 6mg  Walnuts 5mg |
| **Copper** |  |  | Aged cheese (average) 1mg |  |  |  |  | Cocoa Powder 3.9 mg  Cashews 2mg  Chestnuts 1.88mg  Pine nuts 1.32mg  hazelnuts 1.3mg |
| **Selenium** |  | Octopus 75µg |  |  |  |  |  |  |
| **Iron** | Turkey 2.5mg  Bresaola 2.4mg | Anchovies 2.8mg | Egg yolk 4.9mg | Soybeans 6.9mg  Chickpeas flour 6.1mg  Peanuts 3.5mg  Beans 3mg | Wheat bran flakes 40mg  Wheat bran 12.9mg  Wheat germ 10mg  Barley flour and flakes 6mg  Oat 4.7mg  Whole wheat 3.6mg  Whole-wheat flour 3mg |  | Green radicchio lettuce 7.8mg  Chanterelle mushrooms 6.5mg  Nettle leaf 4.4mg  Spinach 2.9mg | Aromatic herbs (leaves) 12.5mg  Pistachio 7.3mg  Cashews 6mg  Dark chocolate 5mg  Hazelnuts 3.3mg  Almonds 3mg |
| **Vit. A retinol equivalent (RE)** |  |  | Butter 930 µg  Egg yolk 640 µg “Pecorino” aged cheese 574 µg  Aged cheese (average) 350-400 µg |  | Yams 655 µg | Mango 533 µg  Apricots 360 µg  Papaya 265 µg  Khaki 237 µg  Cantaloupe 189µg | Carrots 1148 µg  Pumpkin 599 µg  Green radicchio lettuce 542 µg  Spinach 485 µg  Chards 263 µg |  |
| **Vit. B6** | Beef 0.66mg  Horse 0.64mg  Pork 0.62mg  Pork ham 0.61mg  Turkey breast 0.61mg |  |  | Lentils 0.54mg | Wheat germ 3.30mg Wheat bran flakes 1.8mg  Cornflakes 1.8mg  Puffed rice 1.8mg  Muesli 1.6mg  Wheat bran 1.38mg  Corn 0,62mg |  | Leeks 0.64mg | Pistachio 1.70mg  Walnuts 0.67mg |
| **Vit B9 ( folic acid)** |  |  | Egg yolk 130µg | Soybeans 370µg  Soy sprouts 172µg  Fava beans 145µg | Wheat germ 331µg  Wheat bran 260 µg  Wheat bran flakes 250 µg  Cornflakes 250µg  Puffed rice 250µg |  | Nettle leaf 220 µg  Asparagus 165µg  Turnip greens 163µg  Endive 156µg  Spinach 150µg  Brussels sprouts 135µg  Broccoli 132µg |  |
| **Vit. B12** |  | Octopus 20µg  Herring 16µg  Sardines 9µg  Mackerel 7µg | Egg yolk 6.9µg |  |  |  |  |  |
| **Vit. C** |  |  |  |  |  | Blackcurrant 200mg  Kiwi 85mg  Papaya 60mg  Strawberries 54mg  Oranges 50mg  Lemons 50mg  Lychee 49mg  Mandarins 42mg  Grapefruit 40mg  Redcurrant 40mg | Nettle leaf 175mg  Yellow peppers 151mg  Brussels sprouts 81mg  Turnip greens 81mg  Savoy cabbage 77mg  Lettuce 59mg  Cauliflower 59mg  Broccoli 54mg  Spinach 54mg |  |
| **Vit. D** |  | Herring 30µg  Anchovies 11 µg |  |  |  |  |  |  |
| **Vit. E** |  |  |  |  | Wheat germ 22mg |  |  | Sunflower oil 49.2mg  Corn oil 34.5mg  Rice oil 32.3mg  Sunflower seeds 26.1mg  Almonds 26mg  Hazelnuts 24.98mg  Extravirgin olive oil 21.42mg  Soybean oil 8.18mg |

**Supplemental Table 2.** Table reporting mean nutrients’ content in 100g of raw, unprocessed food

SOURCES:

<http://www.bda-ieo.it/wordpress/> (accessed on November 14, 2020)

* <https://fdc.nal.usda.gov/index.html> (accessed on November 15, 2020)

** <https://www.monash.edu/medicine/ccs/gastroenterology/prebiotic/faq> (accessed on November 28, 2020)

^┼^ Should be consumed 1-2 serving per week according to EFSA Scientific Committee. Statement on the benefits of fish/seafood consumption compared to the risks of methylmercury in fish/seafood. EFSA Journal 2015;13:3982

^┼┼^ Sousa V, E. Santos E, Sgarbieri V. The Importance of Prebiotics in Functional Foods and Clinical Practice. Food and Nutrition Sciences 2011;2:133-44
